# SARS-CoV-2 pneumonia—receptor binding and lung immunopathology: a narrative review

**DOI:** 10.1186/s13054-020-03399-z

**Published:** 2021-02-08

**Authors:** Maria Clara Saad Menezes, Diego Vinicius Santinelli Pestana, Gustavo Rosa Gameiro, Luiz Fernando Ferraz da Silva, Ėlodie Baron, Jean-Jacques Rouby, José Otavio Costa Auler Jr

**Affiliations:** 1 Anesthesiology and Intensive Care Department, Instituto Do Coração (InCor), Hospital das Clínicas HCFMUSP, Faculdade de Medicina, Universidade de Sao Paulo, Av Dr Arnaldo, Número 455, São Paulo, SP 01246903 Brazil; 2 Department of Pathology, Faculdade de Medicina, Universidade de Sao Paulo, Sao Paulo, SP Brazil; 3 Multidisciplinary Intensive Care Unit, Department of Anaesthesiology and Critical Care Medicine, La Pitié-Salpêtrière Hospital, Assistance-Publique-Hôpitaux-de-Paris, Sorbonne University, Paris, France

**Keywords:** COVID-19, SARS-CoV-2 pneumonia, ACE2, Pathology, Acute respiratory distress syndrome, Immunology, Hypercoagulability

## Abstract

The current pandemic of COVID-19 caused thousands of deaths and healthcare professionals struggle to properly manage infected patients. This review summarizes information about SARS-CoV-2 receptor binding dynamics and intricacies, lung autopsy findings, immune response patterns, evidence-based explanations for the immune response, and COVID-19-associated hypercoagulability.

## Introduction

In December 2019, a new type of human coronavirus (HCoV) was identified [1] and named severe acute respiratory syndrome coronavirus-2 (SARS-CoV-2), due to its similarity to severe acute respiratory syndrome coronavirus (SARS-CoV) [2, 3].

SARS-CoV-2 causes a disease named coronavirus disease 19 (COVID-19), which main symptoms are cough, fatigue, anorexia, myalgias, anosmia, ageusia and diarrhea. Even though most COVID-19 patients have moderate symptoms and a quick recovery, some patients develop COVID 19 acute respiratory distress syndrome (CARDS). In contrast to acute respiratory distress syndrome (ARDS), CARDS is initially characterized by severe hypoxemia associated to relatively preserved lung compliance until the development of more aggressive phases. Patients may present quite clinically comfortable with a "silent hypoxemia" in early stages [4]. Moreover, dissociation between the laboratory values and imaging presentation is not uncommon [5]. CARDS may be presented into two subtypes: type H (high elastance similar to conventional ARDS) and type L (low elastance) and recognizing them based on CT scan characteristics, for instance, may be paramount to provide appropriate care [6]. Most often, type L precedes type H which rarely appears as the primary form of severe SARS-CoV-2 pneumonia. Ventilation-perfusion mismatch with predominantly dead space areas over shunt portions of the lung could be a hallmark of CARDS [7]. A study advocates that a better alveolar recruitment and greater oxygenation is achieved with high PEEP values even in the L subtype with a high risk of haemodynamic compromise and alveolar hyperinflation [8].

Emerging and reemerging viral threats, such as HCoVs, have continued to challenge public health systems and incur economic and social costs to both individuals and countries [9]. Coronaviruses are enveloped non-segmented positive sense RNA viruses [10] that have long been considered inconsequential pathogens. However, in the twenty-first century, two highly pathogenic HCoVs—SARS-CoV and Middle East respiratory syndrome coronavirus—presumably emerged from animal reservoirs to cause global epidemics [11]. Given the high prevalence and wide distribution of coronaviruses, and increasing human–animal interface activities, novel coronaviruses are likely to emerge periodically as a consequence of frequent cross-species infections and occasional spillover events [12, 13]. Virus-induced direct pulmonary cytopathic effects, viral evasion of host immune responses and exuberant inflammatory responses are believed to play major roles in disease severity [14]. Yet, recent studies with humans who had severe SARS-CoV-2 pneumonia suggest that dysregulation of the immune response results in a compromising inflammation leading to CARDS and lethal outcomes [15]. In this review we aim to discuss recent advances in the understanding of SARS-CoV-2 pneumonia pathogenesis.

### SARS-CoV-2 receptor binding

Angiotensin-converting enzyme 2 (ACE2) is a membrane-bound monocarboxypeptidase found ubiquitously in humans and expressed primarily in pulmonary endothelial cells, alveolar epithelial type II cells, heart, intestine and kidney [16, 17]. ACE2 catalytically removes the last amino acid of angiotensin II (Ang-II), thereby generating the vasodilatory, antifibrotic, antiproliferative and antigrowth peptide Ang-(1–7), which counterbalance the vasoactive Ang-II effects. Investigations focusing on ACE2 have revealed a variety of roles not just catalytic but also as an amino acid transporter and a viral receptor [18]. As shown in Table 1, recent studies have demonstrated that ACE2, which is the main entry receptor of SARS-CoV, is also related to SARS-CoV-2 pathogenicity [1, 19].

**Table 1:** SARS-CoV-2 and receptor binding

Mechanisms of SARS-CoV-2 receptor binding
SARS-CoV-2: similar receptor-binding domain structure to SARS-CoV [20]
SARS-CoV-2 uses angiotensin-converting enzyme 2 to target cells [1]
SARS-CoV-2 and SARS-CoV S protein affinity to angiotensin-converting enzyme 2: similar [21] or ~ 10- to 20-fold higher [22]
SARS-CoV-2 S protein: requires transmembrane protease serine 2 for S protein priming [26]
SARS-CoV-2 S protein: furin-like protease recognition pattern [25]
Soluble recombinant human ACE2 can inhibit SARS-CoV-2 infections [27]
COVID 19 patients: Greater number of angiotensin-converting enzyme 2-positive lung endothelial cells compared with uninfected controls [29]

Although SARS-CoV-2 shares similarity with coronaviruses isolated from bats, its receptor binding domain structure is very similar to that of SARS-CoV [1, 2, 20]. ACE2-expressing human airway epithelial cells cultures inoculated with SARS-CoV-2 show cytopathic effects 96 h after inoculation, including lack of cilia beating. In cultures not expressing ACE-2, cytopathic effects were not observed suggesting that SARS-CoV-2 uses ACE2 as a viral entry receptor [2]. Tight binding between SARS-CoV-2 spike (S) protein and ACE2 partially explains the efficient transmission of SARS-CoV-2 in humans. There is evidence that SARS-CoV-2 S protein binds to ACE2 with an affinity that is 10- to 20-fold higher than the affinity between SARS-CoV S protein and ACE2 [21, 22].

The nature of the cell protease that cleaves the S glycoprotein varies according to the coronavirus. There is evidence that SARS-CoV uses the cellular transmembrane protease serine 2 for S protein priming [23]. Recently, evidence was found that SARS-CoV-2 S protein also uses transmembrane protease serine 2 [24]. Additionally, SARS-CoV-2 S-protein sequence has a specific furin-like protease recognition pattern present in the vicinity of one of the maturation sites of the S protein that is absent in SARS-CoV sequences. Furin protease is a proprotein convertase that is responsible for the activation of precursor proteins, such as growth factors, hormones, receptors and adhesion molecules, as well as cell surface glycoproteins of infectious viruses, thereby having the potential to cleave specifically viral envelope glycoproteins, and enhance viral fusion with host cell membrane [25, 26].

**Fig. 1:**
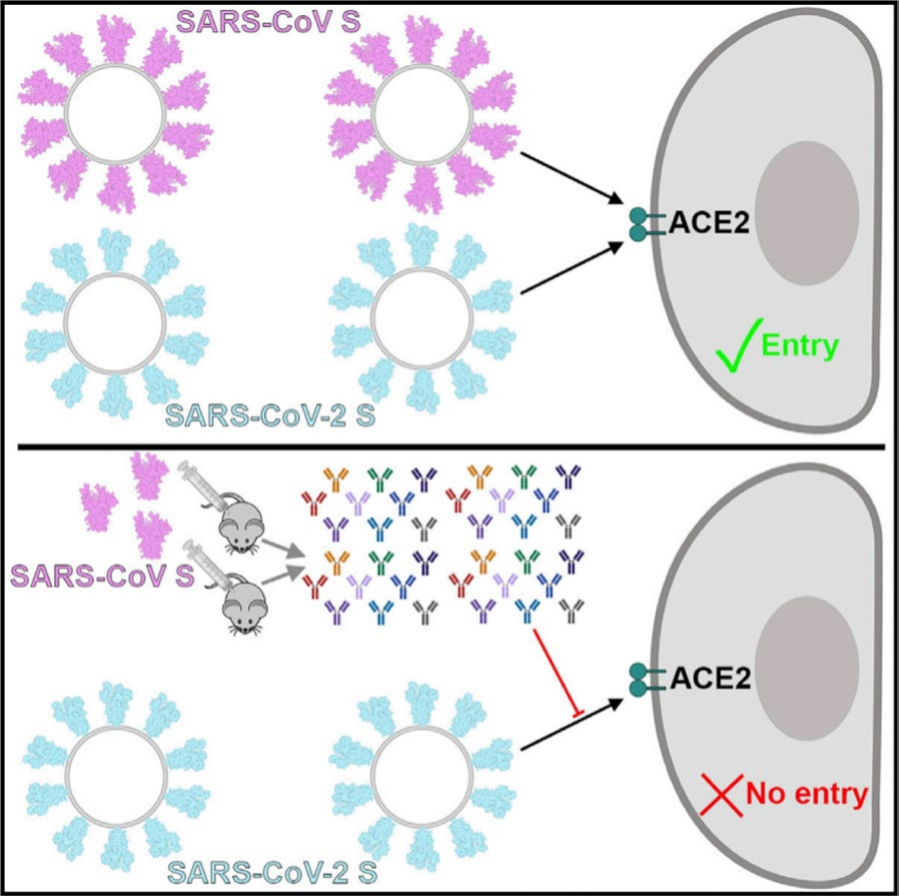
Severe Acute Respiratory Syndrome coronavirus 2 (SARS-CoV-2) binds with high affinity to human angiotensin-converting enzyme 2 (ACE2) and uses it as an entry receptor to invade target cells. Cryo-Electron Microscopy structures of the SARS-CoV-2 spike glycoprotein in two distinct conformations, along with inhibition of spike-mediated entry by SARS-CoV (the coronavirus that emerged in the Guangdong province of China in 2002) polyclonal antibodies, provide a blueprint for the design of vaccines and therapeutics; Permission was granted by Walls et al. (©Elsevier [21]) to reuse this figure

Finally, recombinant human soluble ACE2 molecule—but not mouse soluble ACE2—can significantly inhibit SARS-CoV-2 infections and reduce viral load by a factor of 1000–5000 by reducing viral S protein binding to membrane-bound ACE2. This finding might be used for studying potential therapeutic interventions for COVID-19 [27]. In addition, SARS-CoV polyclonal antibodies inhibit the entry of SARS-CoV-2 into target cells, providing a basis for the design of vaccines (Fig. 1) [21].

### Lung pathology and biomarkers of SARS-CoV-2-induced epithelial and endothelial cells injury

A series of lung autopsies of laboratory confirmed COVID-19 patients have contributed to elucidate the immunopathology behind SARS-CoV-2 pneumonia and the development of CARDS. The main findings are summarized in Table 2.

**Table 2:** Lung autopsy findings from COVID-19 patients

Lung pathology characteristic of COVID-19
Lung oedema [28, 29, 34]
Diffuse alveolar damage [28, 29, 34–38]
Multiple thrombi on the distal lumen of pulmonary vessels [28, 29, 35, 38]
T cells around pulmonary vessels, bronchioles and within interstitium (mainly CD4 and CD8) [28, 29, 31, 34]
Intussusceptive angiogenesis predominating over sprouting angiogenesis [29]
Pulmonary endothelial cells: twice as many angiotensin-converting enzyme 2 when compared to pneumocytes [29]
Pulmonary endothelial cells: disruption of intercellular junctions, swelling, shrinking of capillary lumen, loss of contact with the basal membrane [29, 34]

**Fig. 2:**
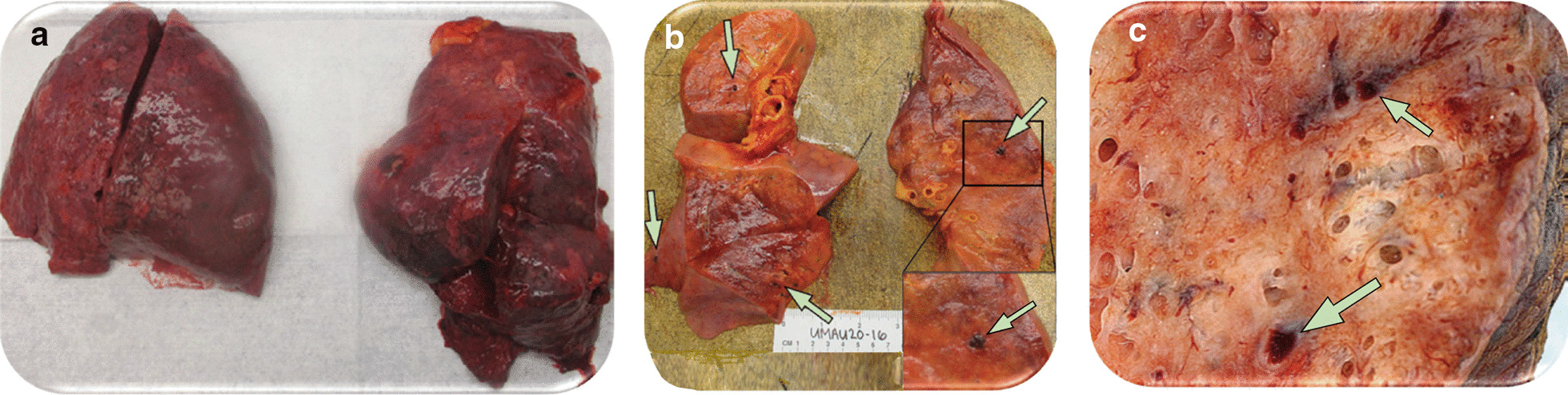
Gross appearance of lungs from two patients who died from severe SARS-CoV-2 pneumonia **a** Lungs with bilateral pulmonary oedema and patches of dark haemorrhage. **b** and **c** Cut sections of lung showing thrombi present within peripheral small vessels (green arrows). Permission was granted by Fox et al. (©Elsevier [28]) to reuse this figure (**a** and **b**) and Permission was granted by Ackermann et al. (©Massachusetts Medical Society [29]) to reuse this figure (**c**)

**Fig. 3:**
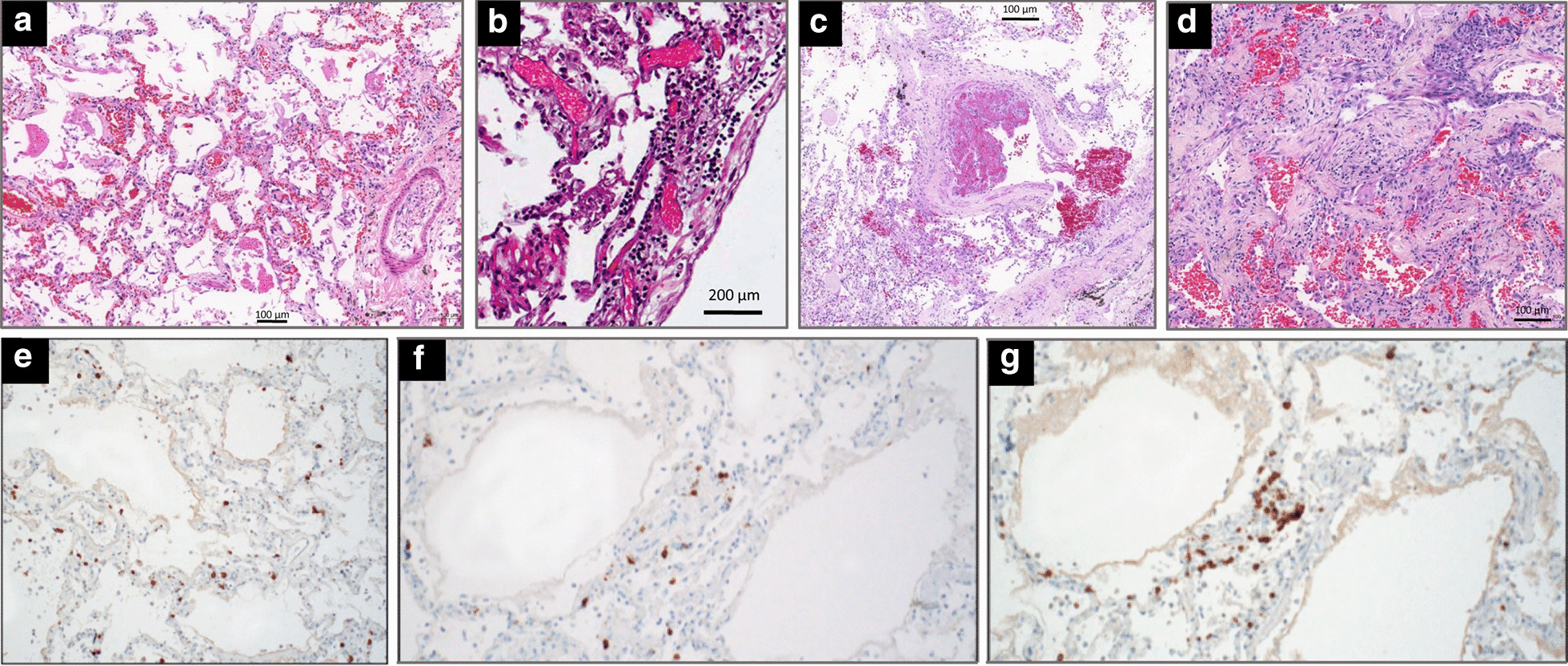
Microscopic findings in the lungs of five patients who died from coronavirus disease 2019. **a** In a 76-year-old man with hypertension who died from a cardiac arrest 10 days after the onset of symptoms (no admission in the ICU), diffuse alveolar damage with vascular congestion, oedema and perivascular lymphocytic infiltration is present; **b** In a 78-year-old man with hypertension, morbid obesity, diabetes type 2 who died from hypoxic cardiorespiratory failure three days after hospital admission and two days of non-invasive ventilation, interstitial pneumonia with perivascular lymphocytic infiltration of interalveolar septa and multifocal endothelialitis is present; **c** In a 63-year-old man without co-morbidity who died from hypoxic cardiorespiratory failure 37 days after onset of symptoms and 26 days after ICU admission and invasive ventilation, partial thrombosis of a pulmonary arteriole with perivascular lymphocytic infiltration is present; **d** In a 64-year-old man without co-morbidity who died from hypoxic cardiorespiratory failure 21 days after onset of symptoms and 15 days after ICU admission and invasive ventilation, diffuse alveolar damage at a proliferative phase is present with collagen plugs deposition in alveolar spaces (hematoxylin staining collagen in light pink and paucicellular areas); **e**–**g** In a 77-year-old man with hypertension and mild obesity, who died on hospital admission six days after onset of symptoms, an interstitial pneumonia is present. T-lymphocytes are highlighted by immunohistochemical stains for CD3 (**e**), CD4 (**f**), and CD8 (**g**). Reproduced from the Department of Pathology of Hospital das Clinicas, Sao Paulo, Brazil (**a**, **c** and **d**) and Permission was granted by Ackermann et al. (©Massachusetts Medical Society [29]) to reuse this figure (**b**) and Permission was granted by Barton et al. (©Oxford University Press [31]) to reuse this figure (**e**–**f**)

Gross examination of lungs from patients with SARS-CoV-2 pneumonia revealed haemorrhagic lung oedema, unfrequently associated with pleural effusions and focal haemorrhages [28, 29]. Multiple thrombi are often visible within the lumen of pulmonary vessels (Figs. 2 and 3c). On light microscopy, perivascular lymphocytic inflammation with preservation of distal airways lumen is the main histological characteristic of SARS-CoV-2 pneumonia at early and late stages (Fig. 3a–d). SARS-CoV-2 pneumonia differs markedly from bacterial ventilator-associated pneumonia where polymorphonuclear leucocytic inflammation centered on an infected bronchiole is the typical histological pattern [30–33]. As shown in Fig. 3e–g, CD8 and CD4-positive T-cells are the predominant lymphocytes identified around pulmonary vessels, bronchioles and within interstitial spaces [28, 29, 31, 34]. Sparse infiltrates of CD3-positive T-lymphocytes can be identified within the alveolar septa with a few CD20-positive B-lymphocytes. Diffuse alveolar damage (DAD), a nonspecific pattern observed in non-COVID ARDS, is also a frequent histological finding [28, 29, 31, 34–38]. During the exudative (acute) phase, DAD is characterized by interstitial oedema, acute and chronic pulmonary inflammation, type 2-pneumocytes hyperplasia, and hyaline membrane formation (Fig. 3d). Lung aeration is preserved at the early phase of severe SARS CoV-2 pneumonia, explaining preservation of respiratory compliance and the characteristic lung ultrasound pattern of diffuse coalescent B lines (Fig. 3a) [33]. During the organizing (healing) phase that is observed after several days in the ICU, the features are similar to those of an organizing pneumonia: granulation tissue (loose accumulations of collagen-embedding fibroblasts and myofibroblasts) and mild chronic inflammation (lymphocytes and plasma cells) (Fig. 3d) [34]; those findings are more rarely found in SARS-CoV-2 patients because lung autopsy is often performed before the patients have entered the healing phase.

**Fig. 4:**
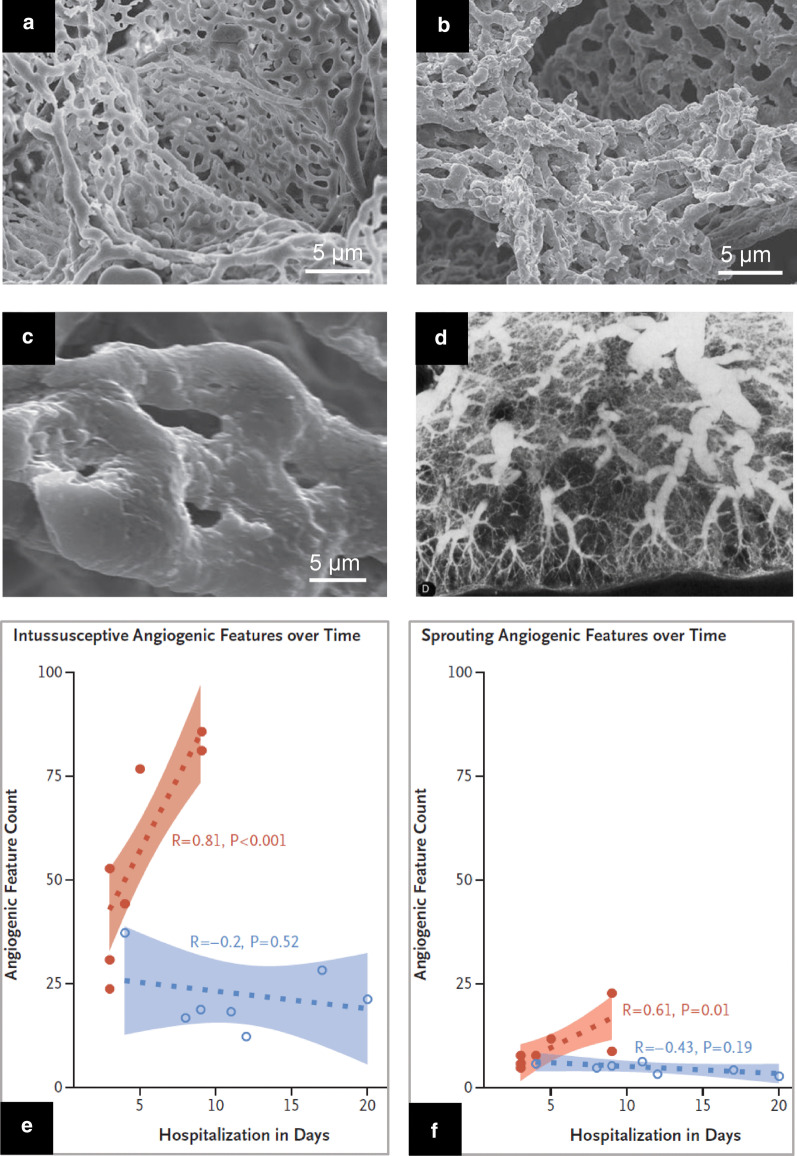
Angiogenesis and neovascularization in patients with severe SARS-CoV-2 pneumonia **a** Electron microcopy showing microvascular corrosion casts from the alveolar plexus of a healthy lung **b** Electron microcopy showing microvascular corrosion casts from the alveolar plexus of a COVID-19 injured lung with substantial architectural distortion **c** Electron microscopy showing pillar localizations (arrowheads) associated with the intussusceptive angiogenesis **d** Postmortem pulmonary arteriogram performed in a patient who died from non-COVID ARDS, 26 days after a massive aspiration. The vascular bed is rarefied and tortuous, suggesting a distorted neovascularization (**e** and **f**) Chronological comparison of intussusceptive and sprouting angiogenesis in lungs from patients with Covid-19 (orange colour) and lungs from patients with influenza A(H1N1) (blue colour) plotted as a function of the duration of hospitalization. In COVID 19 patients, intussusceptive angiogenesis predominates over sprouting angiogenesis and markedly increases with time. Permission was granted by Ackermann et al. (©Massachusetts Medical Society [29]) to reuse this figure (**a**–**c**, **e**, **f**), and Permission was granted by Tomashefski (©Elsevier [39]) to reuse this figure (**d**)

Multiple thrombi are present in the lumen of distal pulmonary vessels including capillaries [28, 29, 34, 35, 38]. These vascular obstructions are frequently observed in non-COVID ARDS [39] and cannot be considered as COVID-19 specific. Associated with vascular thrombi, an early and intense angiogenesis is observed [29]. Compared to patients with severe influenza A (H1N1) pneumonia, angiogenesis in COVID-19 patients is early and massive, resulting in distorted and chaotic alveolar plexus (Fig. 4b), increases with duration of hospitalization (Fig. 4e,f) and occurs predominantly by intussusception (Fig. 4c,e,f). In non-COVID ARDS, tortuous neovascularization is also present at the early and late phases [39] and angiogenesis mechanisms are unknown. Additional studies are required to elucidate how intussusceptive angiogenesis impacts the clinical outcome of COVID-19.

**Fig. 5:**
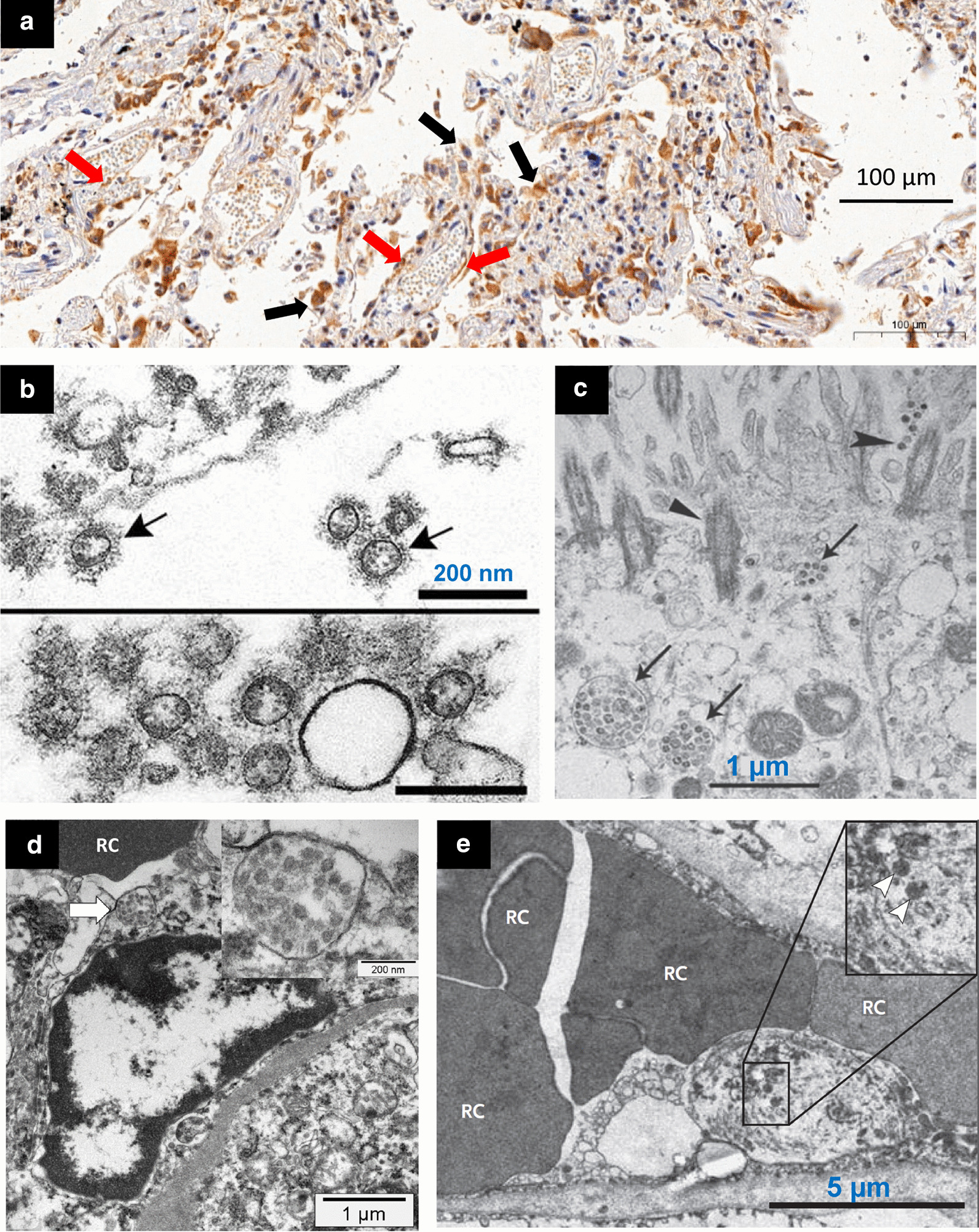
Microscopic histology and electron microscopy showing SARS-CoV-2 in lungs and kidneys of patients who died from COVID 19. **a** In a 76-year-old man with hypertension who died from a cardiac arrest 10 days after the onset of COVID 19 symptoms (no admission in the ICU), positive immunohistochemistry staining for SARS-CoV-2 is present in lung epithelial cells (black arrows) and endothelial cells (red arrows). Immunostaining was performed using a house-made antibody (University of Sao Paulo, Institute of Biomedical Sciences), using a 1:50 dilution, and revealed with 3,3′-Diaminobenzidine staining system **b** Alveolar space containing extracellular SARS-CoV-2 (arrows) with prominent surface profections (bottom: cluster of virions) **c** Extracellular SARS-CoV-2 particles (arrows) present in the airway epithelial and cilia (triangles). **d** An activated glomerular endothelial cell containing a vesicle close to the luminal border with virus-like particles (arrow and insert), adjacent to an erythrocyte (RC). **e** Injured endothelial cell of a pulmonary capillary containing SARS-CoV-2 (arrowheads). Swelling of the nucleus and cytoplasm partially obstructs the capillary lumen. Permission was granted by Martines et al. (©Centers for Disease Control and Prevention [38]) to reuse this figure (**b**), Permission was granted by Zhu et al. (©Massachusetts Medical Society [2]) to reuse this figure (**c**), Permission was granted by Menter et al. (©Public License (Creative Commons) [35]) to reuse this figure (**d**) and Permission was granted by Ackermann et al. (©The New England Journal of Medicine [29]) to reuse this figure (**e**)

SARS-CoV-2 can be directly visualized by electron microscopy [2, 28, 29, 35, 38–43] or evidenced on histologic slices by immunostaining [28, 29, 31, 36, 38, 40, 42]. A note of caution should be added: using electron microscopy, viral particles can be confused with cross sections of rough endoplasmic reticulum [40–42]. Viral particles are not isolated and free in the cytoplasm, but multiple, inside membrane-bound cisternae located within the Golgi area of the rough endoplasmic reticulum. SARS-CoV-2 particles are found in alveolar type II cells with apparent viral cytopathic effect consisting of cytomegaly, and enlarged nuclei with bright, eosinophilic nucleoli [28, 38, 43], in distal airway epithelial cells [2, 38], in pulmonary [29] and renal [35, 40, 44] endothelial cells [Fig. 5]. There are strong arguments to think that SARS-CoV-2 predominantly infect endothelial cells. In addition to the perivascular accumulation of lymphocytes, pulmonary endothelial cells express twice as many ACE2 receptors for viral entry than pneumocytes [29]. Pulmonary and renal endothelial cells are frequently and morphologically injured with disruption of intercellular junctions, cell swelling, shrinking of the capillary lumen, and a loss of contact with the basal membrane, all findings consistent with a central role of endothelial cells in the vascular phase of COVID-19 [29].

Immunostaining demonstrates a prominent expression of SARS-CoV-2 Rp3 NP protein on alveolar epithelial cells, another evidence of direct infection by SARS-CoV-2 [43]. Finally, SARS-CoV viral particles and viral genome have been detected in monocytes and lymphocytes [45]. However, this finding has not yet been confirmed in SARS-CoV-2 infection. Viral injury of epithelial and endothelial cells observed in CARDS is indirectly confirmed by the increase in specific biomarkers. Increase in surfactant protein D plasma level, a biomarker of alveolar type II-pneumocyte injury, is associated with the development of CARDS and macrophage activating syndrome in critically ill patients with severe SARS-CoV-2 pneumonia [46]. Surfactant protein D level is also negatively correlated with PaO_2_/FiO_2_ ratio in those patients, suggesting that surfactant deficiency resulting from injured type II-pneumocytes may contribute to the development of atelectasis and hypoxemia. Unfortunately, the soluble form of the receptor for advanced glycation end product (sRAGE), a well-established biomarker of alveolar type I-pneumocyte injury in non-COVID ARDS [47], has not yet been reported in CARDS patients. It would be interesting to compare plasma and bronchoalveolar lavage sRAGE levels in critically ill patients with CARDS and non-COVID ARDS and assess whether the sRAGE level would allow to separate focal from nonfocal CARDS phenotypes and allow personalized mechanical ventilation [48]. Increase in Angiopoietin-2, soluble E-selectin, and intercellular adhesion molecule 1 plasma levels, all considered as biomarkers of endothelial injury, was predictive of CARDS and admission to the ICU [49, 50]. Interestingly, levels of Angiopoietin-2 were negatively correlated with pulmonary compliance in patients on mechanical ventilation. Very likely, viral infection of endothelial cells triggers high permeability type pulmonary oedema and diffuse alveolar damage, resulting in impaired respiratory mechanics [49]. Similarly, high initial plasma levels of intercellular adhesion molecule 1 were associated with severe forms of SARS-CoV-2 pneumonia, and significantly decreased with recovery, suggesting an alleviation of endothelial cell injury [50].

### Circulating lymphocytes and neutrophils during SARS-CoV-2 pneumonia

Lymphocytes and neutrophils are involved in SARS-CoV-2-induced lung injury. Lymphocytopenia was present in 83.2% of 1099 patients with COVID-19 on admission [51]. Moreover, lymphocytopenia prevalence is higher in ICU than in non-ICU patients (85 vs 54%) and is a risk factor for CARDS [12, 44, 52]. Decrease in lymphocyte count mainly concerns CD8 [53], CD4 and CD3 T-cells [15].

Neutrophilia is also a common finding in severe COVID-19 and is considered a risk factor for CARDS and death [15]. Neutrophils’ antimicrobial and inflammatory functions are mediated by an armamentarium of proteins stored in granules and by the formation of neutrophil extracellular traps [54]. The toxic nature of these traps may pose, however, a threat to highly vascularized tissues such as the lungs. A cell-intrinsic program modifying the circulating neutrophils’ proteome and reducing the neutrophil extracellular traps-forming capacity, protects the lungs against neutrophil-induced inflammatory injury [55]. Finally, there is also evidence of neutrophils immunometabolic reprogramming in COVID-19 patients with increased cytosolic pyruvate kinase muscle, HIF-1α and lactate [56].

### Cytokine responses during SARS-CoV-2 pneumonia

Cytokines are a broad category of small proteins (< 40 kDa) that are produced and released for cell signaling and immunomodulation [57]. An effective and well-coordinated immune response is the first line of defense against viral infections, whereas supraphysiologic immune response can cause organ damage.

The term ''cytokine storm'' is used to express the exuberant inflammatory response observed in severe viral infections [58]. Cytokine storm syndrome is a hyperinflammatory state characterized by fulminant multi-organ failure and elevation of cytokine levels [59]. In COVID-19, the immune response is characterized by high plasma levels of interleukins (IL-6, IL-2), interferons (IFN-y,) chemokines (CXCL10, CCL2, CCL3), growth factors (granulocyte colony stimulating factor) and tumor necrosis factor (TNFα). A high level of IL-10 has also been reported; however, the level of IL-10 was lower in patients with severe COVID-19 when compared to patients with mild COVID-19 [52, 53, 60, 61]. The cytokine profile in the serum is summarized in Table 3.

**Table 3:** Cytokine profile during SARS-CoV-2 pneumonia

Cytokine response during SARS-CoV-2 pneumonia
Severe COVID-19: higher IL-2, IL-7, granulocyte colony-stimulating factor, IP-10, macrophage inflammatory protein1 and tumor necrosis factor-α [44, 52]
Severe COVID-19: lower IL-10 when compared to stable COVID-19 [56]
IL-2, IL-4, IFN-γ and TNF-α: maximum serum level in severe SARS-CoV-2 pneumonia 3–6 days after the disease onset [53]
IL-6: sustained increase and started decreasing around 13–16 days in severe SARS-CoV-2 pneumonia [53]
Cytokine profile in COVID-19 patients resembles haemophagocytic lymphohistiocytosis [69]
Cytokine profile in COVID-19 ARDS less exuberant than in hyper inflammatory phenotype of non-COVID ARDS [72]

IL-6 plays a pivotal role in promoting the inflammatory response observed in severe SARS-CoV-2 pneumonia [62]. The baseline IL-6 plasma level is correlated with pneumonia severity and extension of computed tomography (CT) opacities [63]. Significant decreases in IL-6 and CT opacities are associated with patient’s recovery, whereas time-dependent increase in IL-6 predicts mortality [63–65]. Tocilizumab, a humanized monoclonal antibody, specifically designed to bind soluble receptors for IL-6, could be a therapeutic option for treating severe CARDS [66]. However, a recently published randomized controlled trial did not show any reduction in disease aggravation, admission to the ICU and mortality [67]. Of note, IL-6 is involved not only in the activation of the immune system but also in regenerative processes (anti-inflammatory properties) [68].

Finally, the cytokine profile in COVID-19 patients resembles haemophagocytic lymphohistiocytosis (HLH) syndrome [69]. The cytokine profile of HLH is characterized by high levels of IFN-γ, TNF-α, IL-6, IL-10, and IL-12 [70], a similar pattern to what is found in severe COVID-19 [52, 53, 60, 61]. Other cardinal features of HLH such as cytopenias and hyperferritinemia are also a common finding in severe COVID-19 [51, 61]. HLH is an aggressive and life-threatening syndrome of excessive immune activation. The hyperinflammatory/dysregulated immune state is thought to be caused by the absence of normal downregulation by activated macrophages and lymphocytes causing an excessive cytokine production by macrophages, natural killer cells, and cytotoxic lymphocytes [71].

A note of caution must be added. The relevance of the cytokine storm to COVID-19 pathogenesis has been criticized. There is evidence that the cytokine profile in CARDS is less exuberant when compared with previous cohorts of patients with non-COVID ARDS and the median IL-6 level is 10- to 200-fold lower in CARDS when compared to the hyperinflammatory phenotype of non-COVID ARDS [72]. As a consequence, it is highly likely that the “cytokine storm” is observed in high inflammatory phenotypes of CARDS and is not a characteristic of SARS-CoV-2 pneumonia.

### Possible causes of the “cytokine storm” in severe COVID-19 patients

The immune response seen in critically ill COVID-19 patients is characterized by lymphopenia, neutrophilia and the ''cytokine storm'', which mechanisms are incompletely understood. The host and viral mechanisms associated with SARS-CoV-2-induced immune response are summarized in Table 4.

**Table 4:** Host and viral mechanisms of SARS-CoV-2-pneumonia-associated pathogenesis

Propositions of host and viral mechanisms of SARS-CoV-2-associated pathogenesis		
Direct viral induced pathology	Innate immune responses	Adaptive immune responses
- ACE2 impaired function	- Cytokine overproduction	- Impaired anti-inflammatory properties
- Endothelial dysfunction	- Neutrophilia	- Excessive cytokine production
- Denudation of the airways		- Amplification of local and systemic inflammation

The previously mentioned interaction of SARS-CoV-2 with ACE2 per se may be a primary step in the development of an exuberant lung inflammatory response. ACE2 cleaves Angiotensin I into Ang-(1–9) (an inactive peptide) which is converted to Ang-(1–7) (a peptide with vasodilatory properties), counterbalancing the effects of Ang-II. When ACE2 is attached by SARS-CoV-2 S protein, its intracellular domain induces down-regulation of ACE2 activity, promoting a shift towards the downstream pathway of Ang-II (activation of AngII/angiotensin type 1 receptor axis). Consequently, Ang-II/angiotensin type 1 receptor axis activation leads to glycoprotein 130-mediated activation of signal transducer and activator of transcription 3. This cascade, associated with direct stimulation of pattern recognition receptors, promotes an intense activity of nuclear factor kappa B, which in turns generates increased transcription of IL-6, and triggers the cytokine storm [18, 73].

Ang-(1–7) interacts with mitochondrial assembly receptor (in bronchial smooth muscle and epithelium) initiating an intracellular cascade that yields inhibition of the p38 mitogen-activated protein kinase and nuclear factor-kappa B pathways, ultimately leading to decreased levels of proinflammatory cytokines (such as IL-6, TNF-a, and IL-8) and decreased expression of leukocyte extravasation factors (like intercellular adhesion molecule-1 and vascular cell adhesion molecule-1). Ang-(1–7) also modulates the activities of the extracellular-signal-regulated kinase 1/2 pathway, which modulates the production of IL-10, making the downregulation of ACE2 even worse. Moreover, another function of ACE2 is to cleave terminal residue of [des-Arg9]-bradykinin (BK), a known pulmonary inflammatory factor. [des-Arg9]-BK is a constituent of the kinin-kallikrein system, which acts via BKB1 receptor (BKB1R) and bradykinin B2 receptor (BKB2R). BKB1R expression is modulated by inflammatory cytokines (i.e. IL-1ß and TNF-α via nuclear factor-kappa B activity) and its downstream effect promotes neutrophil migration to pulmonary tissue (via chemokine C-X-C motif chemokine 5), fibroblast growth factor-2 expression, and increased IL-1β and monocyte chemotactic protein 1 levels. BKB2R is stimulated by bradykinin and doesn't appear to be involved in the major events of SARS-CoV-2 pneumonia. In contrast, BKB1R as an important pathway by which down-regulation of ACE2 leads to inflammation [74].

Lymphopenia, characterized by a reduction in peripheral CD4 and CD8 T-cells, is also a prominent feature of severe COVID-19 [64]. As mentioned above, SARS-CoV-like viral particles and SARS-CoV RNA were isolated from peripheral T-lymphocytes [45]. As the receptor-binding domain from SARS-CoV and SARS-CoV-2 shares a lot of similarities [20], it is reasonable to hypothesize that SARS-CoV-2 can directly infect T-cells. This finding associated with other mechanisms such as cell death induced by Fas and Fas ligand interaction and TNF-α-related apoptosis-inducing ligand axis [75], could contribute to the lymphopenia. Although there is a decrease in the absolute count of CD8 and CD4 T-lymphocytes, those cells are found in an overactivated state, harboring high concentrations of cytotoxic granules able to induce severe immune injury [36]. On the other hand, the loss of CD4 T-lymphocytes may cause inflammation as a consequence of impaired production of anti-inflammatory cytokines [73].

Neutrophils and macrophages may also play a role in cytokine overproduction. In influenza infection, there is evidence that lung epithelial cells, macrophages, and dendritic cells all express cytokines via activation of pattern recognition receptors including toll-like receptors (3, 7 and 8) retinoic acid-inducible gene I, and the nucleotide-binding oligomerization domain-like receptor family members [76]. Further studies are needed to assess whether such mechanisms play a role in COVID 19.

Another source of activation of the innate immune response is the endothelialitis evidenced in autopsies from COVID-19 patients [29, 40]. It is possible that as in influenza infection, once the fragile endothelial layer is broken, cytokine and viral antigen exposure can amplify inflammation, with endothelial cells as a major source of pro-inflammatory cytokines [77]. Accordingly, Li et al. hypothesized that upon this barrier breakage monocytes and neutrophils can migrate to the infection site to clear alveolar exudates with virus particles and infected cells, resulting in a loop of uncontrolled inflammation [78].

Finally, the complement system also stands as a pathway contributing to the cytokine storm [74]. Viral activation of complement normally occurs through each one of the three axes: classical, alternative, and lectin pathways. Some of the downstream products of these pathways directly enhance the production of cytokines. The aflatoxin C5a can induce the release of TNF-α; C5b-C9 complex stimulates the secretion of IL-6 from vascular smooth muscle cells, and C3a works as a stimulus to the production of IL-1, IL-6, and TNF-α. Recent data suggest that SARS-CoV-2 is able to cause an aberrant activation of the complement cascade via viral nucleocapsid protein binding to mannose-associated serine protease-2; this interaction promotes activation of mannose-binding lectin, which leads to downstream stimulation of the complement cascade.

Outlining the importance of the cytokine storm control, the randomized multicenter controlled RECOVERY study demonstrated that a daily dexamethasone dose of 6 mg for 10 days reduces day-28 mortality by 15% in patients mechanically ventilated for a severe SARS-CoV-2 pneumonia [79]. A similar benefit is also found in non-COVID ARDS [80].

### COVID-19-associated hypercoagulability

Severe SARS-CoV-2 pneumonia is associated with an increased risk of thromboembolic events, compared to the regular population and to non-COVID-19 ARDS patients [81].

Most of the observed complications are pulmonary embolisms and circuit occlusions during continuous renal replacement therapy or extracorporeal membrane oxygenation. Arterial complications such as strokes, myocardial infarction, renal and mesenteric infarction have also been reported [81]. Elevated levels of anti-phospholipid antibodies have been associated with such events [81]. Laboratory analysis consistently exhibits elevated serum levels of D-dimer and fibrin/fibrinogen degradation products, with mild elevated or normal values of prothrombin time, activated partial thromboplastin time, and platelet count. Serum levels of D-dimer > 2 mg/L are predictive of mortality in critically ill patients with severe SARS-CoV-2 pneumonia [82]. Relatively few cases of disseminated intravascular coagulation have been reported [81], suggesting specific mechanisms for COVID-19-associated hypercoagulability differing from those involved in non-COVD ARDS coagulopathy.

At least four causative mechanisms are suspected: activation of coagulation cascade by the cytokine storm (with involvement of IL-1, IL-6, and tissue factor), impaired functioning of the fibrinolytic system (due to increased release of plasminogen activator inhibitor-1 with a decreased activity of urokinase-type plasminogen activator), inflammation-induced endothelial injury and extensive activation of platelets by pro-inflammatory cytokines and exposition to damaged endothelium [83].

**Fig. 6:**
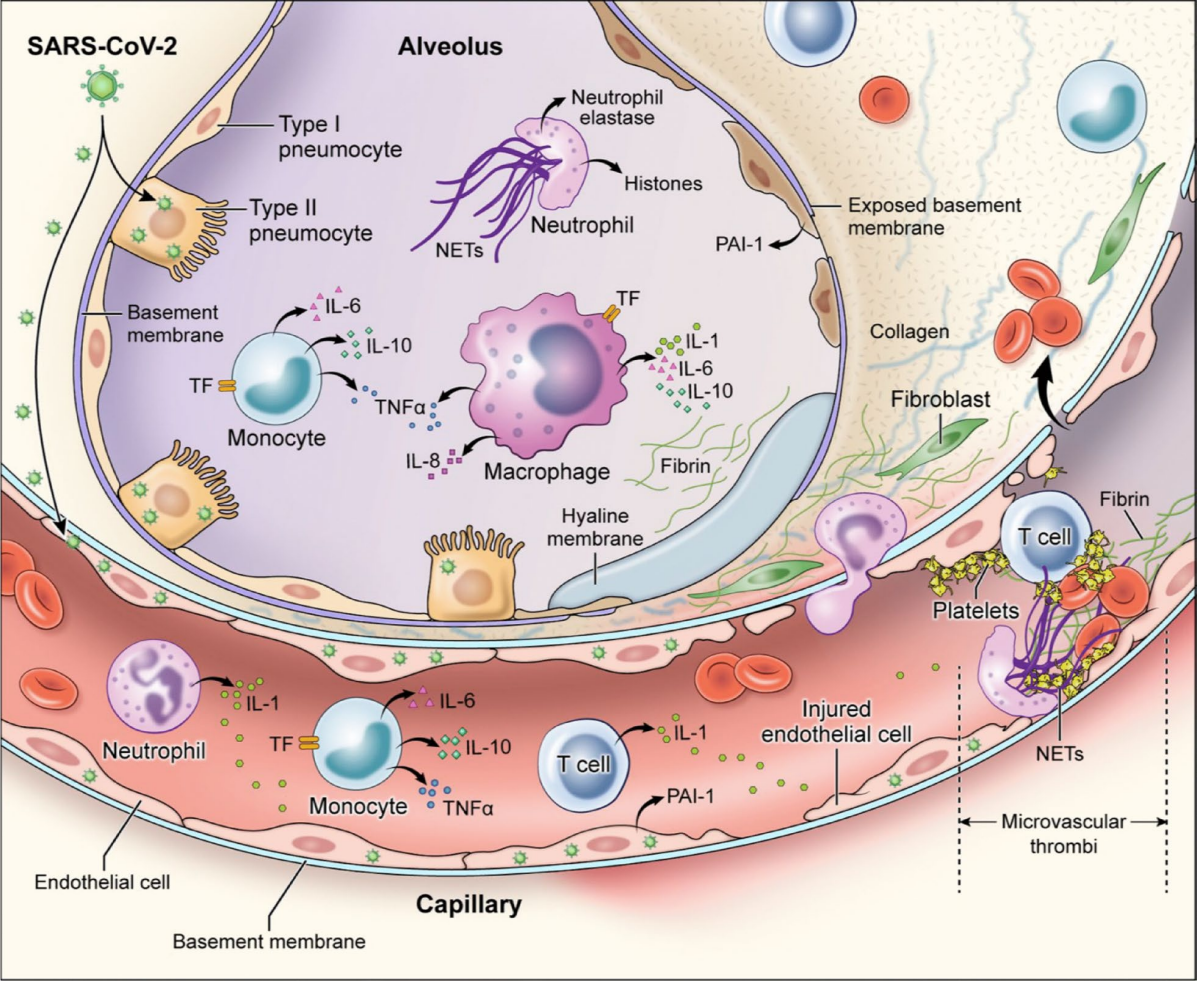
Mechanisms of coagulopathy in patients with SARS-CoV-2 pneumonia: direct infection of type II pneumocytes and endothelial cells, leading to increased barrier permeability; inflammatory activation of T cells, neutrophils, monocytes, macrophages, and platelets resulting in exuberant inflammatory cytokine release, monocyte-derived TF and PAI-1 expression; development of microvascular and macrovascular thrombi composed of fibrin, NETs, and platelets. IL = interleukin; NETs = neutrophil extracellular traps; PAI-1 = plasminogen activator inhibitor-1; TF = tissue factor; TNF-α = tumor necrosis factor-alpha. Permission was granted by Colling and Kanthi (©Public License (Creative Commons) [82]) to reuse this figure

As shown in Fig. 6, macrophages and neutrophils play important roles in the pathogenesis of lung capillary thrombosis. High levels of macrophage inflammatory protein, monocyte chemotactic protein 1 and interferon-inducible protein 10 in the alveolar space as well as the presence of intravascular, extravascular, and intraalveolar neutrophil extracellular traps are illustrative of the importance of these cells in the development of the lung pathologic findings. Moreover, it is possible that SARS-CoV-2 infection is characterized by a direct association between hypercoagulability and inflammation: thrombin binding to proteinase-activated receptor-1 promotes further inflammation by increasing levels of cytokines. Murine models demonstrated decreased inflammation after use of proteinase-activated receptor-1 antagonists [84].

In critically ill patients with SARS-CoV-2 pneumonia, the use of low molecular weight heparin might be associated to a survival benefit, possibly due to a mixed anti-inflammatory/anticoagulation effect [82, 85]. Therefore, some important guidelines recommend use of prophylactic dose low-molecular-weight heparin or unfractionated heparin in all COVID-19 patients requiring hospital admission [86, 87]. Other data encourage the use of therapeutic anticoagulation in patients who met the criteria for sepsis-induced coagulopathy [81]. However, more research is needed to establish indications, safety, and efficacy parameters of thromboprophylaxis.

## Conclusions

The pandemic of COVID-19 forced the scientific community to provide a fast response and a considerable amount of new data have been released since January 2020. The present article identified important pathophysiological landmarks of severe SARS-CoV-2 pneumonia. The binding of SARS-CoV-2 S protein to ACE2 is the main mechanism by which the virus invade target cells. SARS-CoV-2 infects predominantly endothelial cells of pulmonary vessels and capillaries because they express a high density of ACE2 receptors, creating a genuine pulmonary endothelialitis with high permeability-type pulmonary oedema, multiple vascular thrombosis, and neovascularization resulting from predominant intussusceptive angiogenesis. COVID-19 is an interstitial pneumonia characterized by the lung accumulation of lymphocytes around pulmonary vessels associated with lymphocytopenia, predominating on CD4 and CD8 T-cells. In severe forms, there is a cytokine storm resulting from interaction of SARS-CoV-2 with ACE2, overactivation of CD8 T-lymphocytes, loss of CD4 T-lymphocytes, impaired production of anti-inflammatory cytokines, and viral activation of complement through its classical, alternative, and lectin pathways. Last but not least, severe SARS-CoV-2 pneumonia is associated to systemic hypercoagulability resulting from SARS-CoV-2-induced endothelial injury, activation of platelets by exposition to damaged endothelium, activation of coagulation cascade by the cytokine storm, and impaired functioning of the fibrinolytic system. The exact mechanisms by which SARS-CoV-2-induced hypercoagulability remain incompletely elucidated and more research is necessary. The recent demonstration that dexamethasone reduces mortality in mechanically ventilated patients with severe SARS-CoV-2 pneumonia outlines the importance of elucidating pathophysiological mechanisms.

## Data Availability

Not applicable.

## Abbreviations

ACE: Angiotensin converting enzyme
ACE-2: Angiotensin converting enzyme 2
Ang: Angiotensin
ARDS: Acute respiratory distress syndrome
BK: Bradykinin
CARDS: COVID-19 acute respiratory distress syndrome
COVID-19: Coronavirus disease 19
DAD: Diffuse alveolar damage
GCSF: Granulocyte colony stimulating factor
HCoV: Human coronavirus
HLH: Haemophagocytic lymphohistiocytosis
ICU: Intensive care unit
IFN: Interferon
IL: Interleukin
MERS-CoV: Middle East respiratory syndrome coronavirus
SARS-CoV: Severe acute respiratory syndrome coronavirus
SARS-CoV-2: Severe acute respiratory syndrome coronavirus 2
S: Spike
TNF: Tumor necrosis factor
